# Variables associated with the oral impact on daily performance of adults in the state of São Paulo: A population-based study

**DOI:** 10.1371/journal.pone.0203777

**Published:** 2018-09-13

**Authors:** Giovana Renata Gouvêa, Jaqueline Vilela Bulgareli, Luciene Luvizotto David, Gláucia Maria Bovi Ambrosano, Karine Laura Cortellazzi, Luciane Miranda Guerra, Antonio Carlos Frias, Marcelo de Castro Meneghim, Antonio Carlos Pereira

**Affiliations:** 1 Department of Community Dentistry, University of Campinas, Piracicaba Dental School, Piracicaba, São Paulo, Brazil; 2 Department of Community Dentistry, Dental School, USP—University of São Paulo, São Paulo, SP, Brazil; Griffith University, AUSTRALIA

## Abstract

Objective: This study aimed to assess the oral impact on daily performance and its association with sociodemographic characteristics, tooth pain, need for prosthesis, and periodontal disease of adults in the state of São Paulo, Brazil. Methods: This was a cross-sectional epidemiological study with secondary data obtained from the Oral Health Conditions Project– 2015 conducted in 163 municipalities in the state of São Paulo with the participation of 17,560 individuals. This study evaluated adults in the age-range between 35–44 years (n = 5,855), selected by means of probabilistic cluster sampling in two stages. The outcome variable was the OIDP (Oral Impacts on Daily Performances), obtained by using this instrument to assess daily activities (eating, speaking, oral hygiene, relaxation, sports practice, smile, study/work, social contact, and sleep). The independent variables were collected and grouped into three blocks: Block 1 (sex, age group, and ethnic group); Block 2 (household income and education); and Block 3 (tooth pain, need for prosthesis, bleeding, calculus, and periodontal pockets). A hierarchical multiple logistic regression analysis was performed considering the complex cluster sampling plan. Each observation was assigned a specific weight, depending on the location, which resulted in weighted frequencies adjusted for the effect of outlining. Results: the female sex (p<0.0001), ethnic group black/mulatto (p<0.0001), low household income (p = 0.0112), up to 8 years of education (p<0.0001), tooth pain (p<0.0001), presence of bleeding (p<0.0001), and presence of periodontal pockets (p<0.0001) had greater oral impact on daily performance. Conclusion: sociodemographic characteristics, tooth pain, and presence of periodontal disease were associated with oral impact on daily performance of the adult population in the state of São Paulo, Brazil.

## Introduction

Oral diseases have a negative influence on the quality of life [1–2]. Tooth pain and tooth loss cause restrictions on function, lead to discomfort, and hinder food consumption [3], while periodontal changes, such as bleeding and dental calculus, impact the appearance, self-esteem, and even social relationships of individuals. [4].

The results of epidemiological oral health surveys conducted in Brazil in 2010, and in the state of São Paulo in 2015 [5], respectively, have indicated that in adulthood, 83% and 74% of individuals had some problem related to periodontal disease; and some type of prosthesis was needed in 69% and 52% of the cases. Tooth pain was mentioned by 27% and 40% of the adults surveyed. Innovatively, these surveys incorporated the collection of information that enabled analysis of the oral impact on daily performance of Brazilians. These data, associated with the socioeconomic data collected, enabled a set of analyses of the social inequalities related to the subject.

Studies have shown that aspects such as sex, income, ethnic group, and education were associated with the oral impact on daily performance [6–7]. As regards sex, the oral health-related problems are perceived differently by men and women [8]. Individuals with lower income and fewer years of education also presented higher probability of reporting psychosocial alterations in daily life when compared with individuals with high income and more years of education [9–11].

Thus, to understand the factors related to oral diseases it is important to consider the impact of psychological and social factors, since the evaluation instruments and indices most used for determining the actions, strategies, and programs in oral health show strong influence of the biomedical model [12].

The instrument Oral Impacts on Daily Performances (OIDP) is based on the conceptual framework of the International Classification of Impairments, Disabilities, and Handicaps of the World Health Organization [13]. It was developed to measure the oral impacts on physical, psychological, and social aspects of everyday life in the feeling-state dimensions [14] and is particularly significant for use in developing countries [15].

Thus, determining the influence and impact on the performance of daily activities is essential for planning, organization, implementation, and evaluation of the oral health services and programs, since the aspects considered in this evaluation are not only biological and measurable, but also concern the individuals’ self-perception.

Accordingly, this study aimed to assess the oral impact on daily performance and its association with sociodemographic characteristics, tooth pain, need for prosthesis, and periodontal disease of adults in the state of São Paulo, Brazil.

## Materials and methods

This was a government sponsored study in the state of São Paulo conducted from January to September 2015 [5]. The authors were responsible for coordinating the population survey, data collection and carried out the primary investigations of this study.

To make inquiries about the database, all researchers may send an email to apereira@fop.unicamp.br and are asked to please include the following information: name, institution and reason of interest. The data are available at Piracicaba Dental School website http://w2.fop.unicamp.br/sbsp2015/ or Figshare public data repository–License CC BY 4.0 with DOI: 10.6084/m9.figshare.5286025.v1.

The sample size was calculated by using the following mean values: dental caries; prevalence of periodontal conditions; prevalence of use and need for dental prostheses with the respective standard deviations, acceptable error margins (ε), design effects (deff = 2), and non-response rates (NRR = 30%) of the diseases.

Subjects were chosen by conglomerate/cluster sampling in two stages, with probabilities Proportional to the Population Size (PPS), taking into consideration the sample weight and effect of design on the respective stages of the draw. The State of São Paulo was stratified into six macro-regions termed “domains,” and in each domain, 33 municipalities were drawn, termed Primary Sampling Units (PSUs), with exception of the Metropolitan Region of the Capital, where 12 municipalities, in addition to the capital, were drawn. The draws were performed with PPS in each municipality. In the second stage, 390 census sectors (Secondary Sampling Units or SSUs) were selected (two sectors for 178 cities and 36 sectors for the city of São Paulo). The exhaustion technique was used with a minimum sample size for each primary sampling unit, in which all households in the census tract were visited following the planned route, and the individuals of the study age groups were examined. As it was not possible to carry out examinations in all the randomly selected cities (PSUs) in the respective domains, and in some of the census tracts, the sampling fractions were corrected taking into account the non-response rates for each of the stages of the draw. Absentees and those who refused to participate in the study were excluded, totaling 17,560 people examined in 163 cities, of whom 6,051 were adults aged 35–44 [5].

A total of 253 work teams participated in this study. Each team was composed of a dentist who performed the oral exam and applied the questionnaires; and an oral health assistant who took notes of information during the exam. The teams were trained in a workshop with duration of 16 hours, in order to discuss the implementation of the work steps, the assignments of each participant, and ensure an acceptable degree of uniformity for codes and criteria. The examiner calibration process lasted at least 24 hours. The training and calibration processes of the dental teams (dentists and assistants) were conducted by the gold-standard examiner to achieve standardization and agreement among the dental teams. During this phase, examiners studied the codes and criteria, and discussed clinical diagnosis in order to reach an acceptable level of inter-rater agreement statistic. The minimum acceptable inter-examiner kappa value for each examiner, age group, and issue studied was equal to 0.65 [16]. The mean Kappa value for periodontal disease was 0.76 [5].

In this study we evaluated the clinical conditions of periodontal disease and need for prosthesis, tooth pain, socioeconomic characteristics, and oral impact on daily performance. Previously trained and calibrated dentists performed the exams and applied the questionnaires at the participants' homes. To evaluate periodontal conditions, need for prostheses and tooth pain, the codes and criteria recommended by the World Health Organization [17] were used, with the necessary adaptations observed relative to the oral health conditions in the Brazilian population [16].

The OIDP (Oral Impacts on Daily Performances) [14,18,19] was considered the dependent variable. This instrument consisted of nine questions concerning daily life activities, namely: eating, speaking, oral hygiene, relaxation, sports practice, smiling, studying/working, social contact, and sleep. Each item was preceded by the question "Some people have problems that may have been caused by the teeth. Of the situations below, which apply to you, in the last six months?". The options of answers were: no (code 0); yes (code 1); The simple count of scores was obtained through nine variables (yes/no). The OIDP was dichotomized into *with impact* (for answer ''yes'' to at least one question) and *without impact* on daily performances.

**Fig 1:**
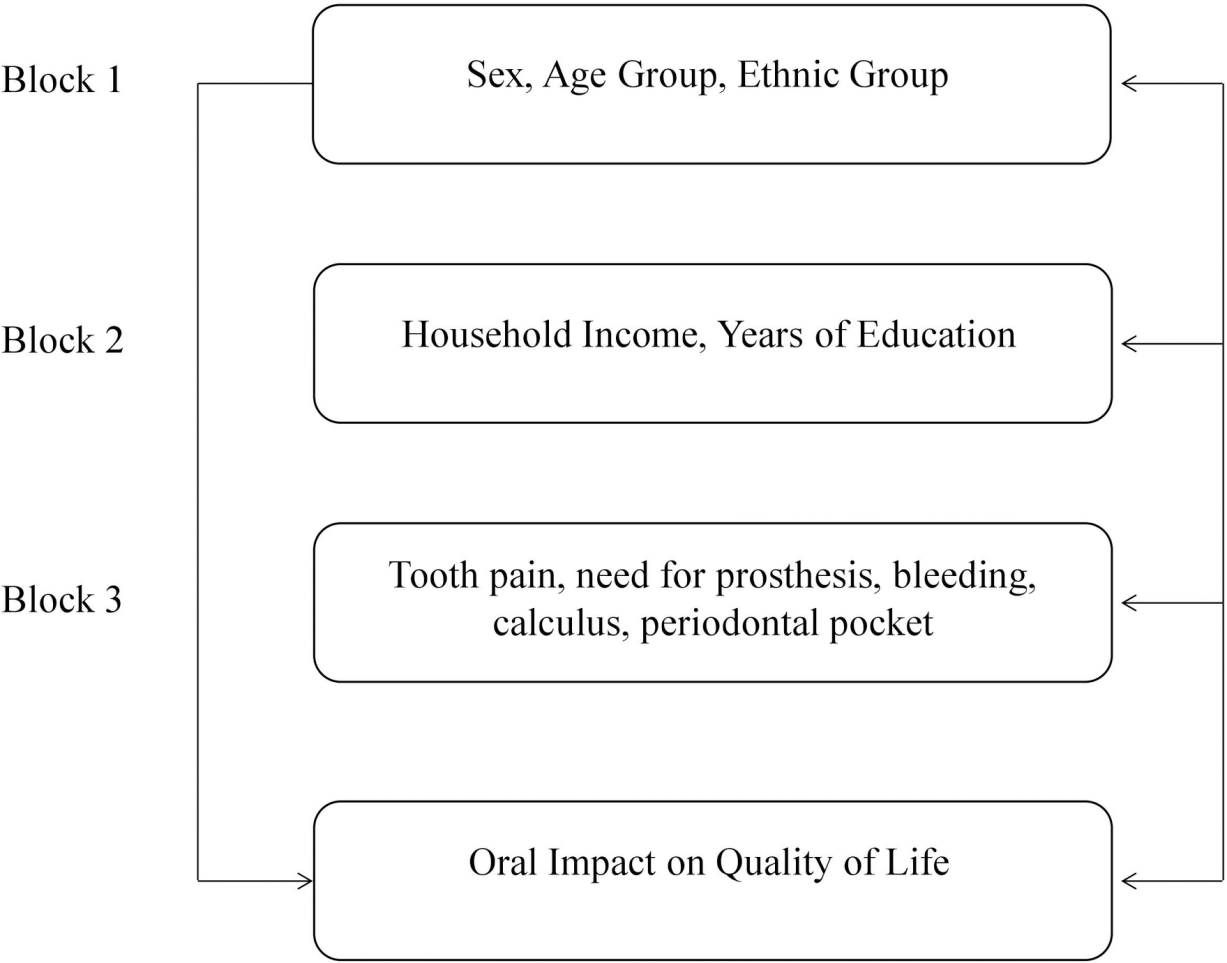
Conceptual model to study the association between independent variables and oral impact on daily performance (adapted from Peres et al., 2013 [1]).

The independent variables were grouped into three blocks: Block 1 included the variables sex, age group, and ethnic group. Block 2 included the variables household income and education. Block 3 included reported tooth pain, need for prosthesis, bleeding, calculus, and periodontal pocket. The variables were organized according to the conceptual model adapted from Peres et al. [1] in which the author uses a hierarchical modeling for multiple analyses. The independent variables were introduced at levels from the most distal to the most proximal ones in relation to outcome (Fig 1).

Initially a descriptive analysis was performed to show the frequencies of oral impact on daily performance according to the variables analyzed. For the data analysis, the association between the OIDP and the independent variables was evaluated through a hierarchical multiple logistic regression. Considering the complex cluster sampling plan, the procedures used for data analysis were the PROC SURVEYFREQ and PROC SURVEYLOGISTIC the software Statistical Analysis System (SAS). Each observation was assigned a specific weight, depending on the location, which resulted in weighted frequencies adjusted for design effect. In the multiple logistic regression models we tested the variables with p≤0.20 of each block, and those associated with the OIDP with p≤0.05 remained in the model after adjusting for the variables of the same block and for those that were hierarchically superior.

This was a study that involved secondary data, to which there was unrestricted access to the database of Oral Health Conditions Project– 2015 and was waived by the Ethics and Research Committee of Piracicaba Dental School (University of Campinas) according to Nº. 094/2015. A term of free and informed consent was signed by all persons examined in the survey (5).

## Results

In this study we evaluated sociodemographic characteristics, tooth pain, need for prosthesis, periodontal disease and OIDP in 5,855 adults population aged 35–44 years. The non-response rate was 22%. The difference in the number of participants in relation to the total number of the sample under study was due to the exclusion of 196 individuals who did not answer one or more items of the OIDP (dependent variable).

The oral impact on daily performance was observed in 2,967 (50.57%) individuals. Table 1 presents results for the frequency of the individuals and their distribution in the variables. The majority of the participants were female (67.8%). As regards the ethnic group, 62.0% were white; 41.7% reported a household income of up to USD 454.00. Tooth pain was reported by 31.4% and 52.3% presented need for prosthesis. The presence of bleeding, dental calculus, and periodontal pocket was 42.9%, 56.0%, and 26.1%, respectively. Of the participants, 54.2% were of the female sex; 50.8% in the age group ≥40 years; 57.5% of the ethnic group black/mulatto; 57.4% with household income of up to USD 454.00; 76.2% reported feeling tooth pain in the last six months; 61.1% needed prosthesis and 60.4%, 58.4% and 65.8% presented bleeding, dental calculus, and periodontal pocket, respectively.

**Table 1:** Distribution of frequencies of oral impact on daily performance.

Variable	Category			OIDP			
				Without impact		With impact	
		n	%	Frequency	%	Frequency	%
Block 1							
Sex	Male	1,888	32.2	1,070	56.6	818	43.3
	Female	3,967	67.8	1,818	45.8	2,149	54.2
Age group	<40 years	2,996	51.2	1,482	49.5	1,514	50.5
	≥40 years	2,859	48.8	1,406	49.2	1,453	50.8
Ethnic group	White	3,628	62.0	1,943	53.5	1,685	46.5
	Black/Mulatto	2,227	38.0	945	42.4	1,282	57.5
Block 2							
Household income	Up to USD 454.00	2,197	41.7	936	42.6	1,261	57.4
	> USD 454.00	3,069	58.3	1,635	53.3	1,434	46.7
Years of education	Up to 8 years	2,679	43.66	1,148	42.5	1,531	57.1
	>8 years	3,176	56.34	1,740	54.5	1,436	45.2
Block 3							
Tooth pain	No	3,979	68.6	2,410	60.5	1,569	39.4
	Yes	1,822	31.4	434	23.8	1,388	76.2
Need for prosthesis	No	2,777	47.7	1,681	60.7	1,096	39.9
	Yes	3,056	52.3	1,189	38.91	1,867	61.1
Bleeding	Absence	3,242	57.1	1,822	56.2	1,420	43.8
	Presence	2,435	42.9	965	39.6	1,470	60.4
Dental calculus	Absence	2,498	44.0	1,465	58.6	1,033	41.3
	Presence	3,179	56.0	1,322	41.5	1,857	58.4
Periodontal pocket	Absence	4,197	73.9	2,281	54.3	1,916	45.6
	Presence	1,480	26.1	506	34.2	974	65.8

According to Table 2, female individuals (OR = 1.48 p<0.0001), of the ethnic group black/mulatto (OR = 1.56 p<0.0001); with household income of up to USD 454.00 (OR = 1.15 p = 0.0112); with up to 8 years of education (OR = 1.33 p<0.0001); with tooth pain (OR = 4.40 p<0.0001); presence of bleeding (OR = 1.28 p<0.0001), and periodontal pocket (OR = 1.53 p<0.0001) had higher probability of presenting oral impact on daily performance.

**Table 2:** Crude and adjusted odds ratios between the impact on OIDP and the variables analyzed.

Variable	Category	OR_b_ (95%CI)	p-value	OR_a_ (95%CI)	p-value
Block 1					
Sex	Male	Ref		Ref	
	Female	1.55 (1.38–1.73)	<0.0001	1.48 (1.30–1.68)	<0.0001
Age group	<40 years	Ref		-	
	≥40 years	1.00 (0.99–1.02)	0.7001		
Ethnic group	White	Ref		Ref	
	Black/Mulatto	1.72 (1.58–1.86)	<0.0001	1.56 (1.40–1.74)	<0.0001
Block 2					
Household income	Up to USD 454.00	1.51 (1.30–1.75)	<0.0001	1.15 (1.03–1.28)	0.0112
	> USD 454.00	Ref		Ref	
Years of education	Up to 8 years	1.63 (1.42–1.87)	<0.0001	1.33 (1.18–1.49)	<0.0001
	>8 years	Ref		Ref	
Block 3					
Tooth pain	No	Ref		Ref	
	Yes	5.29 (4.88–5.75)	<0.0001	4.40 (4.04–4.78)	<0.0001
Need for prosthesis	No	Ref		-	
	Yes	2.29 (2.10–2.51)	<0.0001		
Bleeding	Absence	Ref		Ref	
	Presence	1.83 (1.69–2.00)	<0.0001	1.28 (1.14–1.45)	<0.0001
Dental calculus	Absence	Ref		Ref	
	Presence	1.67 (1.49–1.87)	<0.0001	1.14 (1.00–1.30)	0.0571
Periodontal pocket	Absence	Ref		Ref	
	Presence	2.24 (2.07–2.42)	<0.0001	1.53 (1.39–1.68)	<0.0001

ORb (CI95%) = crude odds ratio with the confidence interval; ORa (CI95%) = adjusted odds ratio with the confidence interval.

## Discussion

In this study the prevalence of oral impact on daily performance was 50.7%, corroborating the findings of another study developed in Brazil, which pointed out the prevalence of 48.1% for the age group 35–44 years [16], and in other countries: India with 50.0% of individuals aged 21–24 years [20]; Tanzania with 51% of individuals with mean age of 26.4 years [21]. Differently, in a study with a population based sample of adults aged 21 years and older, developed in England, Wales, and Northern Ireland, only 16.0% reported oral impacts on daily performance [22]. In addition, the first major national survey covering a wide age range of adults in Norway presented impact of 19.0% on individuals aged 25–44 years and 17.9% in the age group 45–66 years [23]. The discrepancy is in line with the very healthy profile of the Norway and United Kingdom populations, both in terms of oral status and a wide range of subjective measures [22–23].

With regard to sex, in the present study, greatest oral impact on daily performances of this study was shown in the adult female population (54.2%). This result corroborated those found in other previous studies [1, 24–25]. The significant difference between men and women could be attributed to the differences in perception of health and of the value of oral health between the two sexes, in addition to hormonal conditions and higher prevalence of systemic diseases that influence women’s oral health.

In this study the ethnic group black/mulatto and low household income showed association with the outcome, corroborating the findings of other studies that observed that sociodemographic disparities were determinant in the experience of oral diseases. Findings in the literature emphasized that the percentage of black individuals who have never been to the dentist reached 24% compared with 14% for white individuals, and that individuals exposed to the marked sociodemographic and geographic inequalities seemed to be more susceptible to oral diseases [6].

Income is an indicator of socioeconomic position that affects oral health services in developing countries. The social, political, and cultural conditions influence the perception of health; therefore, studies have shown that households with low income have worse perception of their health [26–27]. There is evidence that low incomes and lower educational levels are associated with worse dental health [7]. This finding reinforced the results of this study, which associated low income and few years of education with oral impact on daily performance.

One of the most robust findings in the dental literature, which corroborates the results of this study, is the positive association between education and perception of oral health. This relationship is found in many countries, at different educational levels, and in several dental health indicators [9–11]. Education is a measure of knowledge; capacity to turn knowledge into action; mental flexibility; capacity to seek adequate solutions to problems; thus, a low educational level can have a negative influence on oral impact on daily performance [9–10].

However, education and income shape the social inequalities in dental health, also independently of each other, and have different dimensions of disadvantage, making preventive measures more complicated [9]. An investigation based on longitudinal studies has formulated the proposition that education plays a more important role in preventing the emergence of health problems, while income has greater influence on the course or progression of problems [28].

In this study, tooth pain had negative oral impact on daily performance. Pain is a multidimensional phenomenon that can be influenced by different factors and has significant and detrimental impact on daily performance [29]. In the adult population, pain contributes to absenteeism from work [30] and may impact the individuals’ daily activities, economic production, and work. In a study conducted in the United Kingdom [31], the researchers observed that 37.0% of the users who were working went to an oral health service reporting tooth pain, and 20.0% of these workers had to abandon their function to get dental care.

Thus, providing oral health care to adults should be deemed priority with the purpose of preservation of teeth in this age group, as it will be reflected on the health of this population in the future, considering the increase in life expectancy and oral conditions.

In the context of oral conditions, diseases become severe when they result from some long standing oral problems, such as periodontal disease. Brazil's recent epidemiological data showed that 28.6% of the adults presented dental calculus, 19.4% had periodontal pockets, of which 15.2% were superficial and 4.2%, deep; and only 1.7% had no periodontal disease [16]. Severe Periodontitis (SP) was the sixth most prevalent condition in the world. Between 1990 and 2010, the global standardized prevalence of SP was static at 11.2%, the standardized incidence by age in 2010 was 701 cases per 100,000 persons-year. The prevalence increased gradually with age, showing a marked increase between the third and fourth decades of life, that reached a peak incidence around 38 years of age [32].

In this study, individuals with bleeding, dental calculus and periodontal pockets had higher probability of oral impact on daily performance due to the negative effect on self-esteem and interpersonal relations, corroborating the results found in other countries [4,33]. Therefore, policy makers need to be aware of this growing and predictable burden of periodontal disease, primarily due to the synchronicity with the world population growth, associated with the worldwide increase in life expectancy and significant decrease in the prevalence of total loss of teeth in the period between 1990 and 2010 [32].

The main limitation of this study was its cross-sectional nature, preventing the establishment of any type of causal relations, which made it difficult to define whether the associations observed preceded or followed occurrence of the outcome.

In the case of epidemiology and social sciences, controlled randomized experiments are rarely feasible [34]. However, studies of association play an important role. We noted that the results presented here were reliable, as they were obtained in a representative probabilistic cluster sampling of the adult population of the state of São Paulo. Moreover, in the data analysis we used correction by design effect, a procedure that is highly recommended in studies with complex samples.

Thus, knowledge about the adult population’s oral health conditions and their oral impact on daily performance is essential to enable the implementation of services and public policies. This advance is particularly important for the study of inequalities in health, since the aspects considered in this evaluation are not only biological and measurable, but also concern the individual's self-perception. Knowledge about subjective measures–which are complementary to the clinical measures of the oral health status–is considered essential for defining treatment needs. Moreover, they provide information that can be used to make decisions on policies, such as the definition of priorities with reference to investments, dental services, and dental treatment directed to the greater need of the population or to specific groups.

## Conclusion

Sociodemographic characteristics, tooth pain and presence of periodontal disease were associated with oral impact on daily performance of the adult population in the state of São Paulo, Brazil.
